# Statistically Significant Antidepressant-Placebo Differences on Subjective Symptom-Rating Scales Do Not Prove That the Drugs Work: Effect Size and Method Bias Matter!

**DOI:** 10.3389/fpsyt.2018.00517

**Published:** 2018-10-17

**Authors:** Michael P. Hengartner, Martin Plöderl

**Affiliations:** ^1^Department of Applied Psychology, Zurich University of Applied Sciences, Zurich, Switzerland; ^2^Department for Crisis Intervention and Suicide Prevention and Department for Clinical Psychology, University Clinic for Psychiatry, Psychotherapy, and Psychosomatics, Paracelsus Medical University, Salzburg, Austria

**Keywords:** antidepressant, meta-analysis, efficacy, effectiveness, effect size, clinical significance, method bias

Following the publication of a recent meta-analysis by Cipriani et al. (1), various opinion leaders and news reports claimed that the effectiveness of antidepressants has been definitely proven (2). E.g., Dr. Pariante, spokesperson for the Royal College of Psychiatrists, stated that this study “finally puts to bed the controversy on antidepressants, clearly showing that these drugs do work in lifting mood and helping most people with depression” (https://www.theguardian.com/science/2018/feb/21/the-drugs-do-work-antidepressants-are-effective-study-shows). We surely would embrace drug treatments that effectively help most people with depression, but based on work that has contested the validity of mostly industry-sponsored antidepressant trials (3–6) we remain skeptical about antidepressants' clinical benefits. The most recent meta-analysis indeed concludes that antidepressants are more effective than placebo but also acknowledges that risk of bias was substantial and that the mean effect size of *d* = 0.3 was modest (1). Unfortunately, no clarification is given what this effect size means and whether it can be expected to be clinically significant in real-world routine practice. In this opinion paper we therefore ponder over how the reported effect size of *d* = 0.3 relates to clinical significance and how method bias undermines its validity, in order that the public, clinicians, and patients can judge for themselves whether antidepressants clearly work in most people with depression.

## Statistical vs. clinical significance

Based on statistically significant drug-placebo differences, authors commonly conclude that antidepressants are effective regardless of the clinical significance of effect sizes. Cipriani et al. (7) even complained that there was “an undue focus on the binary and polarizing question of clinical significance” (p. 462). However, statisticians repeatedly cautioned that statistical significance does not imply practical relevance (8–10). A statistically significant result neither proves that the null hypothesis is false nor that the alternative hypothesis is true (8, 9, 11). Interpreting a statistically significant drug-placebo difference as evidence that drugs work is therefore a logical fallacy (12). The null hypothesis is always false, as a true null-association between natural variables (i.e., *d* = 0.0) is nearly impossible due to residual confounding and correlational noise (8, 9). The American Statistical Association (10) formally states that “A *p*-value, or statistical significance, does not measure the size of an effect or the importance of a result” and they further emphasize that “Any effect, no matter how tiny, can produce a small *p*-value if the sample size or measurement precision is high enough …” (p. 132). With a total sample size of *n* = 116,477 as in the most recent meta-analysis (1), it is therefore not surprising that any given drug-placebo difference, however small it may be, reaches statistical significance. Thus, since statistical significance does not imply clinical significance (10, 12, 13), readers need to consider what the reported mean effect of *d* = 0.3 practically means.

**Figure 1:**
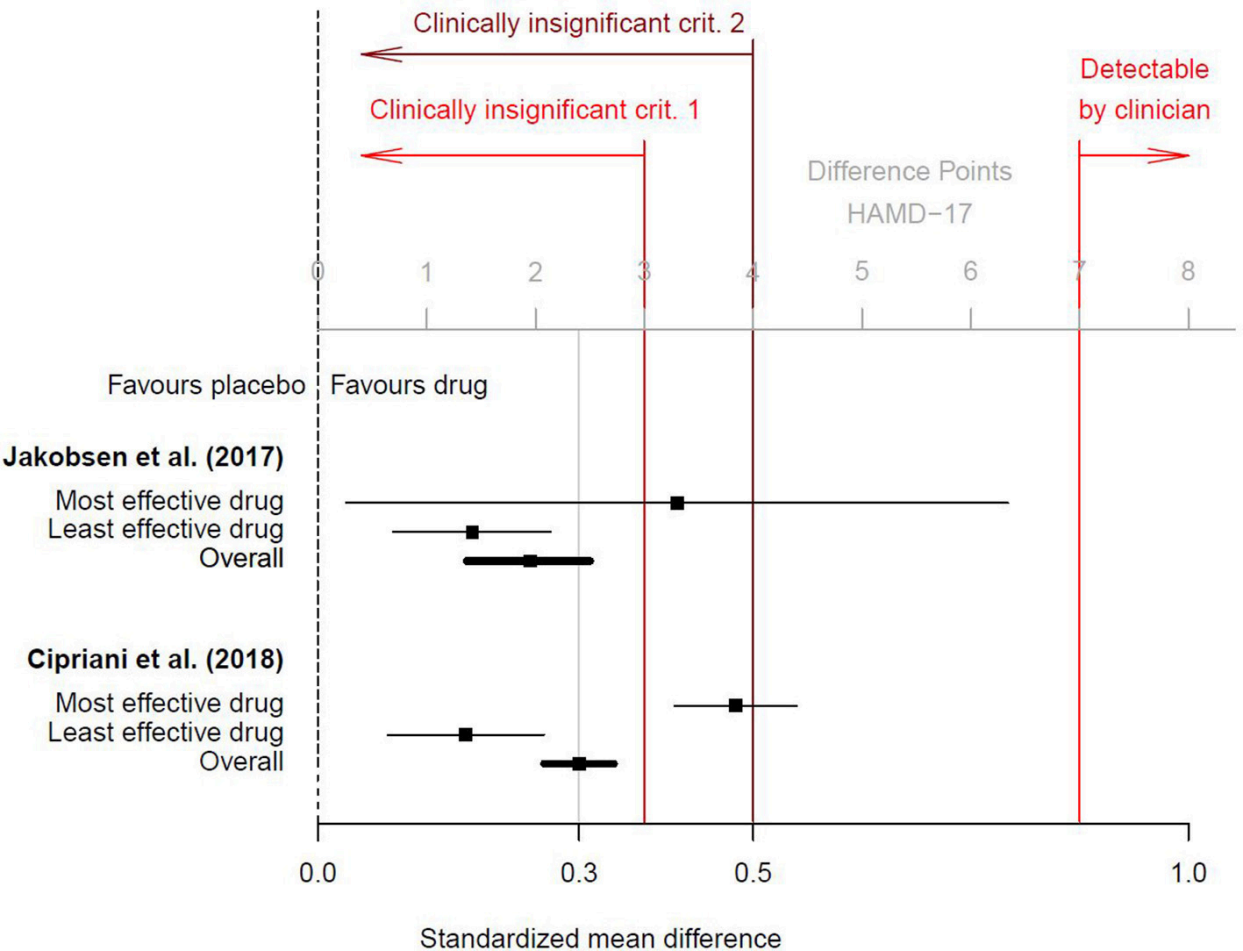
Clinical significance of antidepressants, based on the results of Cipriani et al. (1); additional online information (p. 150) and of Jakobsen et al. (14). Black squares are the standardized mean differences d (drug vs. placebo) for the most and least effective drug and for the overall effect. Horizontal lines are the related 95% confidence intervals. Two conventions for clinical insignificance were used. Criterion 1 was a difference of <3 points on the HAMD-17 scale (corresponding to *d* < 0.4), and criterion 2 was *d* < 0.5. Only differences of at least 7 points on the HAMD-17 scale were found to be detectable by clinicians (16). To transform standardized mean differences into mean point-differences on the HAMD-17 (or vice versa), we assumed a pooled standard deviation of *SD* = 8.0, as suggested by Moncrieff and Kirsch (16) and which conforms to data provided in the online appendix by Cipriani et al. (1).

As shown in Figure 1, this effect size corresponds to approximately 2 points on the Hamilton Rating-Scale for Depression 17-item version (HAMD-17; range 0–52 points), but per convention a difference < 3 points or an effect size *d* < 0.5 (corresponding to < 4 HAMD-17 points) are considered clinically irrelevant (14, 15). Research suggests that drug-placebo differences < 3 points are undetectable by clinicians and that at least 7 HAMD-17 points are necessary for a clinician to detect a minimal improvement in a patient's clinical presentation (16). As a result, the average treatment effect of *d* = 0.3 must be considered undetectable and therefore clinically insignificant in real-world routine practice. Interestingly, a previous meta-analysis by Jakobsen et al. (14) found comparable effect sizes, but the authors defined clinical significance a-priori and therefore questioned the real-world benefits of antidepressants. The effect sizes reported by Cipriani et al. (1) and Jakobsen et al. (14) are plotted in Figure 1.

Here we report Cohen's *d* effect sizes for the sake of completeness and because they are often reported in meta-analyses. However, we emphasize that cut-offs such as *d* = 0.2 (“small” effect size) or *d* = 0.5 (“medium” effect size) are arbitrary and should be interpreted with caution (17). Cohen's *d* is calculated as the mean HAMD-17 difference between treatment groups divided by their pooled standard deviation. When samples are homogeneous and inter-individual variability is low, then the standard deviation is small. All things being equal, the smaller the standard deviation, the larger Cohen's *d*. E.g., a group difference of 2 HAMD-17 points will yield an effect size of *d* = 0.4 when the pooled standard deviation is 5 (2/5 = 0.4), but only an effect size of *d* = 0.2 when the pooled standard deviation is 10 (2/10 = 0.2). The clinical significance of Cohen's *d* further depends on the outcome. A *d* = 0.3 referring to mortality necessarily has more practical relevance than *d* = 0.3 based on subjective (and often transient) symptom ratings.

When based on approximately normally-distributed interval scales, *d* = 0.3 indicates that, first, the outcome of antidepressants and placebo overlap by 88%, second, that only 62% of participants in the antidepressant group score above the mean of the placebo group and, conversely, 38% score below the mean (referred to as Cohen's U_3_), and, third, that if you pick a person at random from the antidepressant group, he/she will have a minor chance of 58% to have the better outcome than a person picked at random from the placebo group (probability of 50% indicates no benefit at all) (17). Finally, assuming a placebo response rate of 35–40% in moderate-to-severe depression (18), based on the Furukawa formula (19), the number needed to treat (NNT) is approximately 9 [see also (20), who calculated a NNT of 8–10 based on the results reported by (1)]. This indicates that, relative to placebo, 9 patients need to undergo antidepressant pharmacotherapy for 1 patient to benefit. In consequence, 8 of 9 patients would equally benefit from an inert placebo pill without risk to eventually suffer from adverse pharmacologic effects (14, 21) and debilitating withdrawal symptoms upon discontinuation of drug treatment (22, 23). A brief synopsis of these findings is that antidepressants might work in a small minority of patients who do not benefit from placebo [see also (24)], but for the vast majority an inert placebo pill that conveys no health risks would work just as well.

## Addressing common objections

A frequently cited paper by Leucht et al. (25) claims that the effect of antidepressants is comparable to that of other medications in general medicine, but note that several general medicine drugs have effect sizes *d* > 0.8, whereas the effect size of antidepressants is *d* = 0.3. Moreover, the general medicine drugs with small effect sizes reported in Leucht et al. (25) were mostly based on objective, severe clinical outcomes such as mortality or cardiovascular events (i.e., “hard” outcomes). Efficacy of antidepressants, in contrast, is exclusively based on subjective symptom ratings (i.e., “soft” outcomes). To provide a fair comparison of the efficacy of antidepressants and general medicine drugs, researchers should base the effect size of antidepressants likewise on a severe clinical outcome such as for instance (fatal) suicide attempts. In that case the effect size of antidepressants would be close to zero and favoring placebo (26–30). This compares very unfavorably to most general medicine drugs.

Another unsubstantiated objection is that the efficacy of antidepressants is poor due to inadequate psychometric properties of the HAMD-17 [e.g., its poor content validity (31)]. We do not intend to defend the validity of the HAMD-17, but instead we want to stress that when the efficacy of antidepressants relies on other outcome measures, effect sizes are not higher. First, when efficacy is based on patient self-reports such as the Beck Depression Inventory (BDI), mean effect sizes are even smaller (i.e., *d* < 0.3) than those based on the HAMD-17 (32, 33). Second, a meta-analysis of all escitalopram trials sponsored by Forest and Lundbeck, which applied the Montgomery-Asberg Depression Rating Scale (MADRS), produced a mean effect size of *d* = 0.32 (24). Third, there is no evidence from clinical trials that antidepressants work when efficacy is based on severe clinical outcomes such as suicide attempts (26–30). I.e., the HAMD-17 is not accountable for antidepressants' poor efficacy.

A third objection is that critics of antidepressants unjustifiably promote psychotherapy although talk therapy is no better than pharmacotherapy. In response to these concerns we would like to state that we have also written about the limitations and biases in psychotherapy research (34). We further agree that in the short-term (i.e., acute treatment), the outcome of psychotherapy and pharmacotherapy is comparable (35). Cuijpers and Cristea (36), two prominent psychotherapy researchers, proposed that enhanced placebo effects could explain the short-term outcome of both pharmacotherapy and psychotherapy. Nevertheless, in the long-term, psychotherapy conveys less physical health risks and its effect on depression (i.e., sustained remission and relapse prevention) appears to be superior to pharmacotherapy according to several meta-analyses of direct comparisons (37–39).

## The efficacy of antidepressants is overestimated

The average treatment effect detailed above, albeit minor, yet is most likely an overestimation due to various systematic biases that inflate the apparent efficacy of antidepressants, including, in particular, unblinding of outcome assessors (3, 36, 40). Treatment effects in antidepressant trials are commonly rated by clinicians who can identify with high accuracy which patients receive the active drug and which inert placebo based on the reporting, or a suspicious lack thereof, of recognizable side effects such as nausea or dry mouth (36, 41). Several lines of evidence suggest that drug-placebo differences might be inflated when efficacy estimates are based on subjective symptom rating-scales such as the HAMD-17.

First, it has consistently been shown that treatment effects are larger when the outcome is rated by unblinded assessors, thus efficacy estimates are inflated due to assessors' treatment expectancies (42–44). Second, when active placebos that mimic common antidepressant side effects are applied instead of inert placebos, the estimated treatment effects are substantially smaller because assessors are more effectively blinded (45). Third, antidepressants' efficacy has been shown to be substantially smaller when estimates are based on patients' self-reported depression symptoms instead of observer-ratings (32, 33), suggesting that patients do not perceive the same benefit as (unblinded) clinicians attribute to the drugs. Fourth, with respect to dropouts due to any reason, which is regarded as an objective measure of real-world effectiveness (46), antidepressants are, on average, not superior to placebo (1, 47). Finally, fifth, evidence for assessor bias was also shown in the most recent meta-analysis, where antidepressants were judged more efficacious when they were novel as compared to when they were older (1). Since a drug does not lose its pharmacologic effect simply because it has been on the market for a few years, this is evidence for a systematic overestimation of novel drugs due to clinicians' treatment expectancies.

Given that the mean drug-placebo difference is only about 2 HAMD-17 points, even a minor bias in symptom-ratings could fully account for antidepressants' treatment effect. Indeed, taking the observer bias into account, Gotzsche (48) calculated that the effect of antidepressants, relative to placebo, is virtually zero (OR = 1.02). Note that there are many more systematic biases than unblinding of outcome assessors that we did not consider here. These include, for instance, the selective inclusion of participants (patients who are known to preferably respond to the experimental drug are included in the trials, while none-responders and patients who experienced bothersome side effects prior to the actual trial are excluded), patient expectancy bias (patients believe that the drugs work, thus producing an enhanced placebo response which takes effect as soon as a patient realizes that he/she receives the active drug), inadequate management of missing data (the common procedure of “last observation carried forward” produces inflated efficacy estimates), and outcome reporting bias (quite often only results for the most convenient outcome are reported and interpreted) (3, 49, 50).

## Conclusions

Contrary to the predominant interpretation we contend that antidepressants do not work in most patients, given that only 1 of 9 people benefit, whereas the remaining 8 are unnecessarily put at risk of adverse drug effects. To be clear, antidepressants can have strong mental and physical effects in some patients that may be considered helpful for some time (51), but there is no evidence that the drugs can cure depression (3, 40, 48). Insomnia, fatigue, loss of appetite, psychomotor agitation, and suicidal acts are recognized depression symptoms (52), but newer-generation antidepressants may cause precisely these symptoms (14, 29, 46, 53). This is not what we would expect from drugs that effectively treat depression. Moreover, emerging evidence from well-controlled long-term pharmacoepidemiologic studies suggests that antidepressants may increase this risk of serious medical conditions (21, 54, 55), including dementia (56), stroke (57), obesity (58), and all-cause mortality (57, 59, 60). Antidepressants may have clinically meaningful short-term benefits in a small minority of patients, but the most recent meta-analytic evidence does not indicate that they work in the majority of patients. A careful re-evaluation of risks and benefits is therefore needed before the controversy about the utility of antidepressants can be put to bed.

## Author contributions

MPH drafted the manuscript. MP contributed significantly in writing and critical revision.

### Conflict of interest statement

The authors declare that the research was conducted in the absence of any commercial or financial relationships that could be construed as a potential conflict of interest.
